# Comparison of two different suction curettage methods in cesarean scar pregnancy treatment

**DOI:** 10.1186/s12884-024-06917-x

**Published:** 2024-10-31

**Authors:** Burak Elmas, Neslihan Ozturk, Emine Kizil, Bergen Laleli Koc, Ugurcan Zorlu, Duygu Tugrul Ersak, Turkan Dikici Aktas, Asuman Erten, Salim Erkaya

**Affiliations:** 1 Department of Gynecology and Obstetrics, University of Health Sciences Ankara City Hospital, Ankara, Turkey; 2 Department of Gynecology and Obstetrics, University of Health Sciences Etlik Zübeyde Hanım Women’s Health Training and Research Hospital, Ankara, Turkey

**Keywords:** Cesarean scar pregnancy, Suction curettage, Ectopic pregnancy, Treatment modalities

## Abstract

**Background:**

Cesarean scar pregnancy (CSP), the incidence of which is increasing, can lead to life-threatening consequences. In this study, it was aimed to compare the results of two different ultrasound-assisted suction curettage (SC) approaches that we applied to endogenous type CSPs in different time periods.

**Methods:**

Patients who were diagnosed with CSP and treated with SC in the early pregnancy service between January 2012 and March 2019 were included in the study. While classical SC was applied until December 2016, patients were treated with SC modified by us after this date. Demographic characteristics, preoperative clinical findings, intraoperative characteristics and postoperative short-term follow-up of these two groups of patients belonging to different time periods were compared.

**Results:**

34 patients were treated with classic SC (Group 1) and 32 patients with modified SC (Group 2). The amount of decrease in Hemoglobin values measured at the sixth hour postoperatively compared to the preoperative period was found to be less in group 2 (1.01 ± 0.67 g/dl) than in group 1 (1.39 ± 0.85 g/dl) (*p* = 0.042). The treatment failure rate was found to be lower in group 2 (*p* = 0.028). According to the results of multiple logistic regression analysis of significant factors associated with treatment outcome, myometrial thickness measurement and the largest gestational diameter measurement were found to be significant independent factors.

**Conclusion:**

In CSP cases, SC procedure with abdominal ultrasonography is an effective and reliable approach. At the beginning of this surgical procedure, if the gestational sac is removed from the uterine wall with the curettage cannula before suction, the success of the procedure will increase even more.

**Supplementary Information:**

The online version contains supplementary material available at 10.1186/s12884-024-06917-x.

## Background

Cesarean scar pregnancy (CSP) is the implantation of the gestational sac to the previous cesarean scar line that has not healed properly and was first described about half a century ago [1]. Although it was a very rare condition in the past, its frequency is gradually increasing as a result of increasing cesarean rates and developments in diagnostic methods over time. Although the incidence is not known exactly, it is observed between 1/2000 and /1800 of all pregnancies, it constitutes 4.2-6.1% of all ectopic pregnancies. It has been reported that the probability of CSP increases 1.15 times in individuals who have given birth by cesarean section at least once before [2, 3]. The diagnosis of CSP is difficult, and the delay in diagnosis may lead to life-threatening consequences such as uterine rupture, massive bleeding, hypovolemic shock, may require hysterectomy, and may cause loss of fertility and additional morbidity in the patient [4, 5].

CSP is divided into two groups according to its growth propensity. In the endogenous type, the gestational sac grows into the uterine cavity or the cervico-isthmic junction. There is a possibility of live birth in this type, however, placental adhesion disorders and concomitant heavy bleeding and the need for hysterectomy may occur. In the exogenous type, the pregnancy grows towards the uterine serosa, which is most likely associated with uterine rupture [6].

Many different methods are used in the treatment of CSP. Expectant approach, double-balloon catheter, application of systemic-local methotrexate, suction curettage (SC), hysteroscopic resection, uterine artery embolization, high-intensity focused ultrasound and hysterotomy (abdominal or laparoscopic excision) are among these methods [2, 7]. Among these approaches, suction curettage is the most frequently applied surgical approach and is the first choice preferred by some clinicians [8–10]. However, it is considered suitable for the endogenous type with a myometrial thickness of 3 mm or more between the gestational sac and the uterine serosa [11, 12]. Laparoscopic removal of the CSP surgically is an appropriate approach for the exogenous type [13]. However, since it is a rare situation, there is no clear opinion about which method should be applied.

In our study, we aimed to compare the results of two different ultrasound-assisted suction curettage approaches that we applied to the endogenous type CSPs in the different time periods and to determine the factors that may be related to the results of these treatment approaches.

## Methods

Patients who underwent SC procedure with the diagnosis of CSP in the Early Pregnancy Service of our hospital, which is a tertiary center between January 2012 and March 2019 were included in this retrospective study. Approval was obtained from the hospital ethics committee for the study (2019/11). This hospital is a localized referral Gynecology and Obstetrics Hospital in Ankara, the capital city of Turkey. It is an important training center where more than 10,000 births and over 2000 gynecological operations are performed annually. All CSP diagnoses were made by trained specialist gynecologists by transvaginal ultrasonography. Godin criteria were used in the diagnosis [14] : (1) history of previous cesarean section, (2) serum beta-human chorionic gonadotropin (Beta-hCG) elevation, (3) empty uterine cavity and cervical canal image in ultrasonographic evaluation, (4) detection of gestational tissue embedded in the scar line, (5) thin myometrial tissue or absence of myometrial tissue between the gestational mass and the bladder.

Patients with known bleeding diathesis, hypertension, diabetes and cardiac problems were not included in the study. Patients whose myometrial thickness (distance between the gestational sac and the bladder) was less than 3 mm in the ultrasonography or who showed invasion into the uterine serosa and bladder were excluded. Patients who received other treatment (such as intracavitary or intramuscular methotrexate) for CSP before SC and patients whose records could not be fully accessed were also excluded from the study.

Between January 2012 and December 2016, the SC approach to eligible CSP patients was standardized in our clinic. For the procedure, the patient was prepared in the lithotomy position under general anesthesia in operating room conditions. After proper site cleaning and cervical dilatation, Carmen cannula 6 was inserted into the cavity under the guidance of abdominal ultrasound. Subsequently, the injector part was inserted and a constant pressure of 300 mm Hg was activated and the four uterine walls were curetted (Group 1). In January 2017 and later, a modification was made to the method. Again, after the cervical dilatation, the carmen cannula 6 is inserted into the cavity under the guidance of abdominal ultrasound to the patient, who is prepared properly in the operating room. The connection between the uterine four walls and the scar pregnancy sac is separated a little with forward and backward movements without “pressure activated”, then a curettage is activated by activating a constant pressure of 300 mm Hg and the curretage is completed (Group 2). The main difference between the classical suction curettage and the modified method we suggest is based on the ultrasound-guided separation of the gestational sac from the uterine wall more gently and then activating the pressure before using pressure before starting the curettage. The process of disrupting the sac without activating the negative pressure and activating the pressure afterwards has been included in our clinical practice, considering that the negative pressure minimizes the damage to the endometrium and myometrium tissues at the first moment (Supplementary File 1). All of the operations were carried out by Senior Prof. Erkaya and Assoc. Prof. Elmas.

Both procedures were applied to patients whose vital signs were within normal limits and hemodynamically stable. The procedure was terminated when the gestational sac disappeared in ultrasonography. After the procedure in both groups, a 16 F urinary catheter was placed in the uterine cavity under the guidance of abdominal ultrasonography, and the balloon was inflated to 30 cc localized to the cesarean scar, and tamponade was applied, and this catheter was removed at the end of 24 h. All patients were informed about the pre-procedure method and possible complications, and gave their consent that they were aware of method and complications and accepted the procedure.

Demographic characteristics, preoperative clinical findings, intraoperative characteristics and postoperative short-term follow-up of these two groups of patients belonging to different time periods were recorded. Successful treatment was defined as disappearance of the CSP sac and a negative serum β-hCG level (< 10 mIU/ml) without the need for additional treatment (medical or surgical) or a major complication such as bleeding, uterine rupture, or hysterectomy. Those who needed additional treatment (medical or surgical) after the procedure and/or the presence of major complications such as heavy bleeding (requiring transfusion), uterine rupture or hysterectomy were classified as unsuccessful treatment.

### Statistical analysis

SPSS 22.0 program was used for statistical analysis. The normal distribution of the data was evaluated with the Kolmogorov Smirnov test, numerical data with normal distribution were shown as mean ± standard deviation, and the difference between the two groups was investigated with the independent t test. Those that did not show normal distribution were shown as the median (minimum-maximum) and the Mann-Whitney U test was used for the difference between groups. Categorical data were shown as numbers (%), and Chi-square test or Fisher’s exact test was used in comparison of groups, depending on their suitability. Multiple logistic regression analysis was applied to identify independent factors that may be associated with treatment outcome. Threshold values for significant independent factors were calculated by receiver operating characteristic (ROC) analysis. *P* < 0.05 was considered statistically significant.

## Results

Initially, 78 CSPs were detected. Six of them were excluded from the study because they were not suitable for the interventional procedure (additional medical problems, unsuitable myometrial thickness, invasion into the uterine serosa, urgent need for laparotomy). Six of them were excluded due to insufficient registration, too. As a result, 34 patients were determined for group 1 and 32 patients were determined for group 2.

The characteristics of these two groups of patients are listed in Table 1. Accordingly, the mean age, gravida, parity and gestational age values of the existing CSP were similar in the two groups. The durations of the groups until the detection of CSP after the last cesarean section and the frequency of Dilation&Curettage (due to early pregnancy loss or voluntary termination of pregnancy) after this C Section were similar. In terms of symptomatology, the groups did not show any difference.

**Table 1:** Characteristics of the groups

	Group 1 (*N* = 34)	Group 2 (*N* = 32)	*P*
Age	33.42 ± 5.09	32.66 ± 6.39	0.596
Gravida	3.85 ± 1.35	3.91 ± 1.78	0.891
Parity	1.85 ± 0.70	1.97 ± 0.93	0.569
Number of C Section	1.71 ± 0.52	1.81 ± 0.74	0.499
Number of C Section ≥ 2	23 (67.6)	20 (62.5)	0.661
Symptomatology Asymptomatic Vaginal Bleeding Pelvic pain	17 (50.0) 13 (38.2) 4 (11.8)	15 (46.9) 12 (37.5) 5 (15.6)	0.898
Gestational age (days)	45.39 ± 9.11	44.09 ± 9.15	0.569
Time since last C Section (months)	23.29 ± 6.15	23.75 ± 10.55	0.830
Presence of D/C after last C/S	4 (11.8)	1 (3.1)	0.357
Anteverted uterus	26 (76.5)	24 (75.0)	0.889
Largest gestational diameter (mm)	26.35 ± 12.65	25.41 ± 8.19	0.721
Myometrial thickness (mm)	4.04 ± 0.35	4.16 ± 0.32	0.196
β hCG value before treatment (mIU/ml)	7247 (431-59705)	4990 (107-70542)	0.739
Presence of fetal heart beat	11 (32.4)	10 (31.3)	0.923
Preop-postop Hg difference	1.39 ± 0.85	1.01 ± 0.67	0.042
Need for Blood transfusion	1 (2.9)	1 (3.1)	0.965
Duration of hospitalization (days)	3.62 ± 3.52	2.53 ± 1.74	0.120
Duration for β Hcg resolution (days)	19.06 ± 2.08	18.65 ± 1.84	0.392
Treatment failure	9 (26.5)	2 (6.3)	0.028
Variables were presented as mean ± standard deviation, median (minimum-maximum) and number (%). C/S: cesarean section, D/C: dilatation curetage; *p* < 0.05 was considered statistically significant.			

When the pre-treatment examination and laboratory findings were examined, no difference was found between the groups in terms of β-hCG level measured before treatment, myometrial thickness, the largest gestational diameter of CSP and the presence of fetal heart beat. The amount of decrease in Hemoglobin values measured at the sixth hour postoperatively compared to the preoperative period was less in group 2 (1.01 ± 0.67 g/dl) than in group 1 (1.39 ± 0.85 g/dl) (*p* = 0.042). Duration of hospitalization and resolution (become negative) of β-hCG values were similar between both groups.

11 (16.7%) patients required additional treatment or procedure and they were classified as unsuccessful treatment. 9 of these patients were in Group 1 and 2 of them were in Group 2. Additional methotrexate treatment was given to 5 of the patients in Group 1, CSP excision was made by laparotomy to 3 of the patients in Group 1 and CSP excision was performed to 1 of them by laparoscopy. One of the patients who underwent laparotomy received 1 unit of erythrocyte suspension intraoperatively. The reason for transfusion was that the preoperative Hemoglobin value was below 10 mg/dl and active bleeding was observed intraoperatively. Additional methotrexate treatment was given to 1 of the patients in Group 2, and excision was performed by laparotomy in the other patient. Intraoperatively, 1 unit of erythrocyte suspension was given to the patient who underwent laparotomy. The reason for transfusion was that the preoperative Hemoglobin value was below 10 mg/dl and active bleeding was observed intraoperatively. There was a statistically significant difference between the two groups in terms of the frequency of unsuccessful treatment (*p* = 0.028) (Table 1). No patient in either group had heavy bleeding, uterine rupture or hysterectomy.

**Table 2:** Comparison of patients according to treatment results

	Successful (*N* = 55)	Unsuccessful (*N* = 11)	*P*
Age	32.62 ± 5.58	35.18 ± 6.23	0.177
Gravida	3.73 ± 1.59	4.64 ± 1.21	0.078
Parity	1.85 ± 0.85	2.19 ± 0.60	0.228
Number of C Section	1.71 ± 0.66	2.00 ± 0.45	0.166
Number of C Section ≥ 2	34 (61.8)	9 (81.8)	0.204
Gestational age (days)	43.95 ± 8.90	48.82 ± 9.30	0.105
Time since last C Section (months)	23.11 ± 8.85	25.55 ± 6.47	0.390
Presence of D/C after last C/S	4 (7.3)	1 (9.1))	0.611
Anteverted uterus	44 (80.0)	6 (54.5)	0.072
Largest gestational diameter (mm)	4.19 ± 0.28	3.65 ± 0.30	< 0.001
Myometrial thickness (mm)	23.79 ± 8.96	36.45 ± 12.50	< 0.001
β hCG value before treatment (mIU/ml)	5276 (107-70542)	9887 (1700–45418)	0.064
Presence of fetal heart beat	14 (25.5)	7 (63.6)	0.013
Group 1 or 2	25 (45.5) / 30 (54.5)	9 (81.8) / 2 (18.2)	0.028
Variables were presented as mean ± standard deviation, median (minimum-maximum) and number (%). C/S: cesarean section, D/C: dilatation curetage *p* < 0.05 was considered statistically significant.			

The comparison of the patients with and without successful treatment is shown in Table 2. Among the parameters measured, the mean myometrial thickness measurement was significantly higher (*p* < 0.001) in the successful treatment group (4.19 ± 0.28 mm) compared to the unsuccessful treatment group (3.65 ± 0.30 mm), while the largest mean gestational diameter measured was smaller (23.79 ± 8.96 mm and 36.45 ± 12.50 mm, respectively; *p* < 0.001). At the same time, fetal heart beat positivity (25.5% and 63.3%, respectively; *p* = 0.013) and the frequency of Group 1 (45.5% and 81.8%, respectively; *p* = 0.028) were higher in those whose treatment was unsuccessful.

**Table 3:** Logistic regression analysis results of factors associated with treatment outcome

	Wald	SE	*p*	Exp (B)	%95 CI for Exp (B)
Myometrial thickness (mm)	8.13	2.39	0.004	0.01	0.00-0.12
Largest gestational diameter (mm)	4.20	0.05	0.040	1.10	1.00-1.20
Presence of fetal heart beat	1.98	1.21	0.159	5.48	0.51–58.33
Treatment Group	2.40	1.47	0.122	9.75	0.55-174.45
*p* < 0.05 was considered statistically significant.					

According to the results of multiple logistic regression analysis of significant factors associated with treatment outcome, myometrial thickness measurement and the largest gestational diameter measurement were found to be significant independent factors (Table 3). The threshold value for myometrial thickness measurement in predicting successful and unsuccessful treatment results according to ROC analysis results is 3.95 mm with 81.8% sensitivity and 80.0% specificity [Area under curve (AUC): 0.906, Standard Error (SE): 0.043, 95% Confidence Interval (CI):0.822–0.990, *p* < 0.001]; The threshold value for the largest gestational sac diameter was calculated as 26.5 mm (AUC: 0.798, SE: 0.082, 95% CI: 0.638–0.957, p: 0.002) with 81.8% sensitivity and 69.1% specificity.

## Discussion

We obtained important results in our study. One of them is that Transabdominal Ultrasound-Guided Suction Curettage (TAUG-SC) is an effective and safe method, especially in hemodynamically stable pregnancies with endogenous type CSP. Secondly, it is the opinion that the failure rate may be less if we start the procedure by removing the gestational sac from the uterine wall. Finally, SC procedure may fail as the gestational sac grows and myometrial thickness decreases.

In the literature, there are studies in favor of SC [8, 15] and against [16, 17] regarding the efficacy and safety of SC in the treatment of CSP. The reason for this diversity lies in the differences in patient selection and application technique. Polat et al. reported that they were able to successfully treat their patients with suction curettage at a rate of 84.2% in < 8 weeks and in the presence of > 2 mm myometrial thickness. They treated 12 of 19 patients with SC only and 4 of them with SC + foley balloon tamponade without any problem and defined the SC method as an effective and safe method in the type of patients they chose [10]. It has been reported that performing the procedure with transabdominal ultrasound would be a good option for effective treatment [8, 11, 15, 18]. However, it has been reported that the blinded SC procedure has high unsuccessful rates and is a method that may cause complications, so it would be an approach that should not be applied [2, 10, 19]. We were able to successfully treat 55 of our 66 endogenous type CSP cases, in which we applied TAUG-SC + foley balloon tamponade, without the need for any additional medical or surgical methods. In addition, we did not detect any major or life-threatening complications in any of the patients. Therefore, we believe that the TAUG-SC + foley catheter tamponade method can be used as an effective and safe approach in primary treatment in hemodynamically stable endogenous type CSP cases.

SC is a low-cost, easy-to-apply method with a low side-effect profile; but it must be done properly. Unfortunately, the ideal SC application method in the presence of CSP has not been defined [20]. While Polat et al. recommended aspiration of the entire uterine cavity with the use of constant pressure (300 mmHg) during SC, they suggested avoiding excessive pressure against the uterine walls, especially the anterior Wall [10]. On the other hand, Weilin C. and Li J suggest that for SC procedure, first aspirate the upper part of the uterine cavity and its posterior wall with a higher pressure (400 mmHg), and then aspirate the lower segment of the anterior wall with a lower pressure (200–300 mmHg) when most of the pregnancy contents are aspirated and the uterus begins to contract [8]. Such differences are mainly based on personal experience and there is no study on their accuracy. As a result of the clinical experience we gained over time, we thought that the low-constant pressure SC procedure after weakening the connection between the gestational sac and the uterine four walls with the help of a cannula placed in the cavity under ultrasound guidance before applying pressure could be more effective and reliable than the unmodified version. The procedure we applied without modification included the SC procedure, which was performed with low pressure by entering the cavity. As a matter of fact, when we examined the results of these two SC procedures, which we compared in our study, we found that the success rate was higher in the newer approach (Group 2) and the decrease in the post-procedure Hemoglobin value was less. These results suggest that although there is no difference between the two methods in terms of need for blood transfusion and significant complications such as heavy bleeding, uterine rupture, and hysterectomy were not observed in either method, the clinical experience we gained over time with the new approach is correct.

It has been reported that SC can be performed successfully if CSP is diagnosed early (before 7–8 weeks of gestation), low β-hCG value (< 17000 mIU/ml) and myometrial thickness is sufficient (> 2 mm) [5, 8, 10]. In our study, although the gestational age and β-hCG values were higher in the unsuccessful treatment group, they did not create a statistically significant difference. This may be a result of the limited number of study groups and although they do not show a statistical difference, it may indicate a clinically important point. As the gestational age progresses, the pregnancy grows, the serum β-hCG level increases and the trophoblastic invasion in the myometrium deepens. Growing pregnancy and increasing myometrial invasion inevitably increase the possibility of failure of the SC procedure. In the presence of decreased myometrial thickness measurement (between gestational sac and uterine serosa) as a result of increased invasion, the risk of complications such as bleeding and rupture due to SC procedure increases [8, 10].

In our study, the largest gestational sac diameter length and myometrial thickness measurement were found to be independent factors associated with the SC procedure, confirming this situation. Increase in gestational sac measurement or decrease in myometrial thickness were determined as factors that increase the probability of unsuccessful treatment. In the literature, different myometrial thickness measurements (between 3 and 4.5 mm) in which the SC procedure has been successfully applied have been reported [8, 9, 15], but no threshold value has been defined. However, there is no information about gestational sac. In our study, the presence of myometrial thickness greater than 3.95 mm was associated with a successful SC procedure with relatively high sensitivity and specificity. Measurement above the gestational sac diameter of 26.5 mm was associated with a failed SC outcome with relatively high sensitivity and low specificity. These findings are the first in the literature, and as with any new finding, they need further evaluation and validation in order to be used in clinical use.

### Strengths and study limitations

We think that our study is important and instructive as it is the first study to compare two different types of SC procedures in the surgical treatment of endogenous CSP. We also think that the presence of similar groups of patients in terms of many factors that may cause heterogeneity in the comparison of both groups, and the fact that both the diagnosis of CSP and the SC procedure for the treatment of CSP are performed in a referral center create confidence in the evaluation of the treatment results. On the other hand, the fact that the data were collected retrospectively and the study population was relatively limited may raise doubts about the reliability of the results. We use the postoperative 6th hour Hemoglobulin value in our hospital routine. If necessary, depending on the patient’s condition, an extra Hemogram test is requested. Since it was a retrospective study, we performed the study with postoperative 6th hour Hemoglobulin test. One of the important limitations of our study is that the amount of bleeding could not be clearly determined. The postoperative 24th hour hemoglobulin value could have provided more valuable results. However, the retrospective design limited our ability to reach the postoperative 24th hour hemoglobulin result in all patients. In future studies on this subject, measuring the amount of actual bleeding and increasing the sample size will make the study even more powerful.

## Conclusion

Abdominal ultrasonography-guided SC procedure followed by foley balloon catheter tamponade is an effective and safe approach in cases of endogenous type, hemodynamically stable CSP. At the beginning of this surgical procedure, if the gestational sac is removed from the uterine wall with a curettage cannula before suction, it seems to be a safer technique that reduces the failure rate of the procedure. It should also be kept in mind that the probability of failure of the SC procedure increases as the gestational sac grows and the myometrial invasion of the sac increases, and as a result, the distance between the sac and the uterine serosa decreases.However, for CSP, which is a rare condition, good quality prospective randomized comparative studies with large participation are needed to confirm this information.

## Electronic supplementary material

Below is the link to the electronic supplementary material.

Supplementary Material 1

## Data Availability

The datasets generated and/or analysed during the current study are not publicly available due [We are a hospital affiliated with the Ministry of Health and respect for the privacy of patients but are available from the corresponding author on reasonable request.
