# Supplementary material for: Comparison of two different suction curettage methods in cesarean scar pregnancy treatment
Source: BMC Pregnancy Childbirth. 2024 Oct 31;24:717. doi: 10.1186/s12884-024-06917-x (PMC11526517; doi:10.1186/s12884-024-06917-x)
Supplement: Supplementary file 1 — Supplementary Material 1 [file 12884_2024_6917_MOESM1_ESM.docx]

**Supplementary File 1:** Diagram of classical curettage treatment and modified curettage treatment

**Group 1. Classical suction curettage**


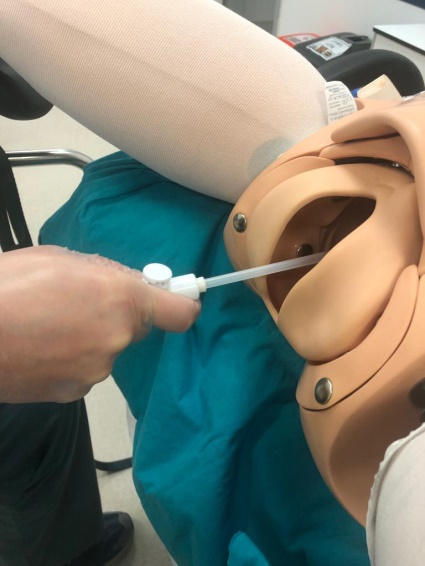


**1.** Carmen cannula 6 was inserted into the cavity under the guidance of abdominal ultrasound.


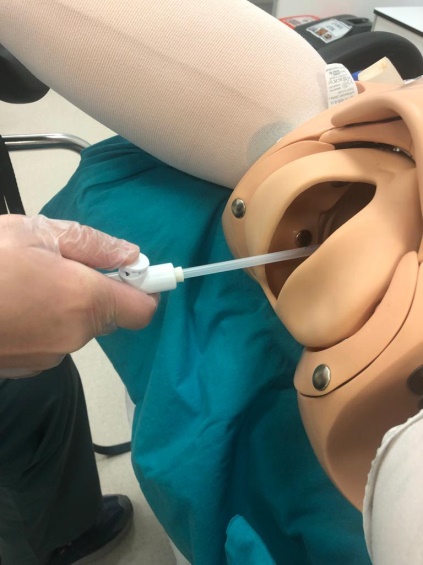


**2.** Subsequently, pressure button unlocked,


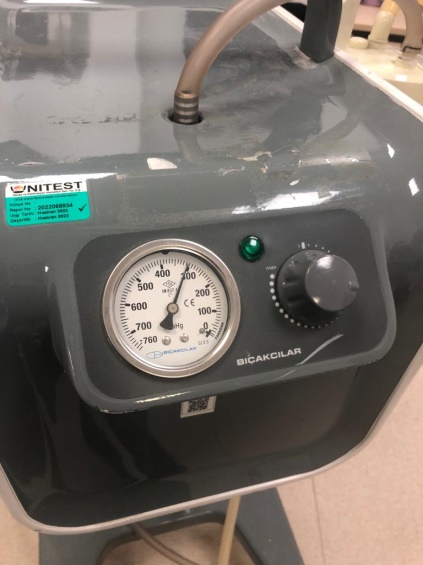


**3.** And a constant pressure of 300 mm Hg was activated.


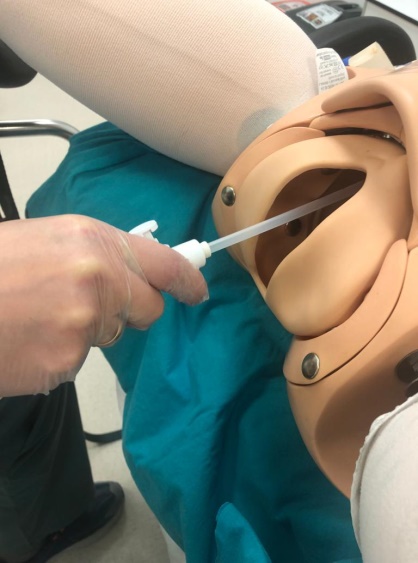


**4.** After activated the suction, the four uterine walls were curetted.


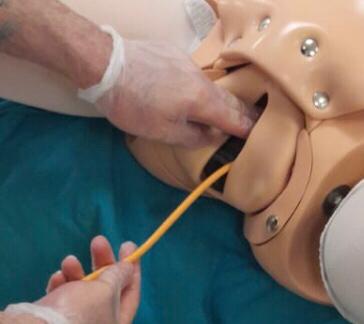


**5.** A 16 F urinary catheter was placed in the uterine cavity under the guidance of abdominal ultrasonography, and the balloon was inflated to 30 cc localized to the cesarean scar, and tamponade was applied, and this catheter was removed at the end of 24 hours.

**Group 2. Modified suction curettage**


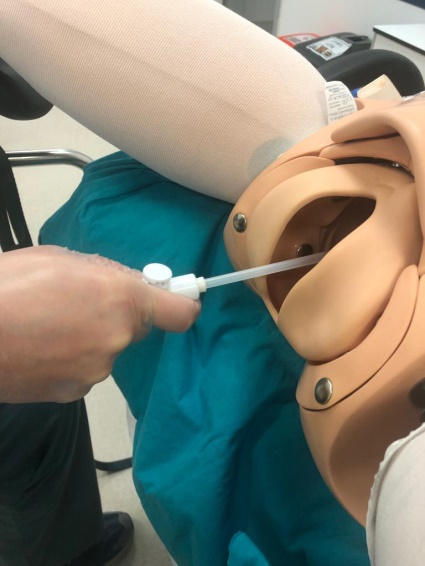


**1.** The carmen cannula 6 was inserted into the cavity under the guidance of abdominal ultrasound.


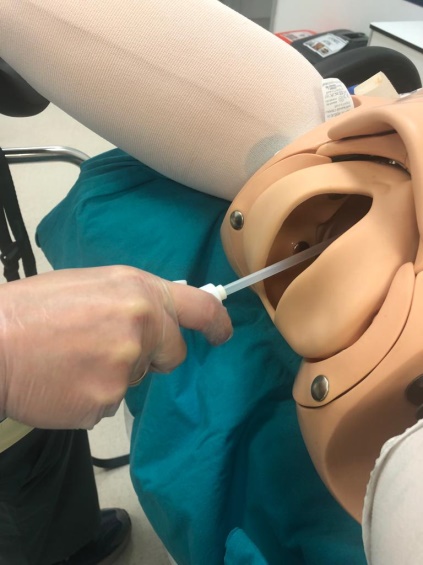


**2.** The connection between the uterine four walls and the scar pregnancy sac was separated a little, with forward and backward movements ,as well as clockwise and counterclockwise movements “without pressure activated" (**pressure button locked off** ).


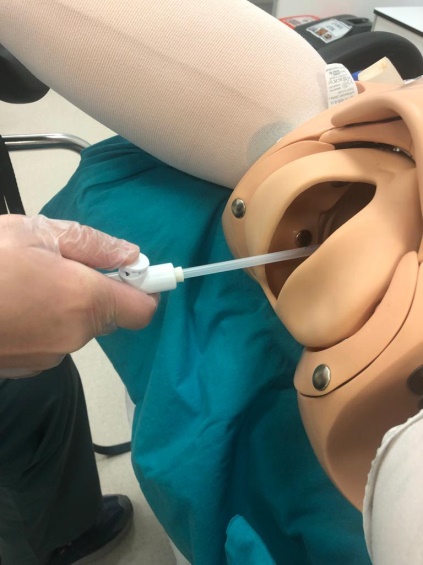


**3.** Then, pressure button unlocked.


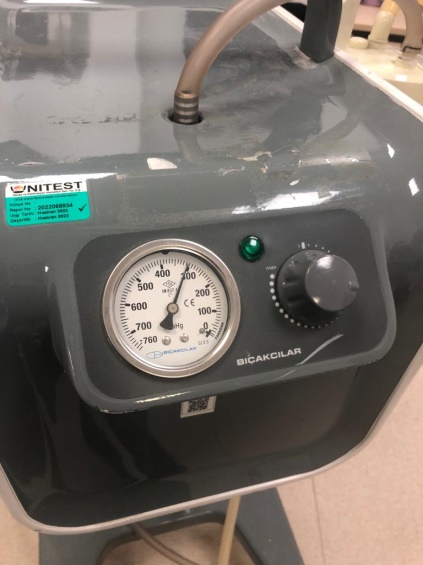


**4.** A constant pressure of 300 mm Hg was activated.


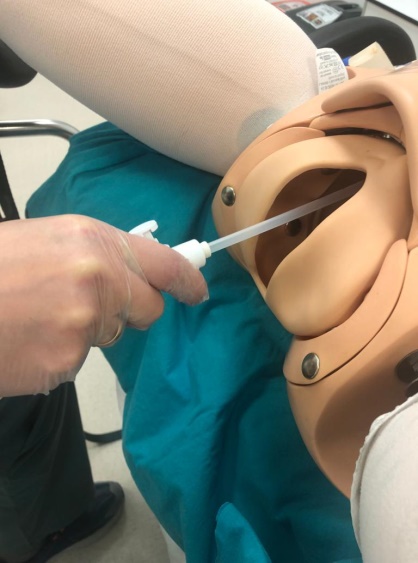


**5.** And the curretage is completed by curettage of four walls, as it was done in classical suction curettage.


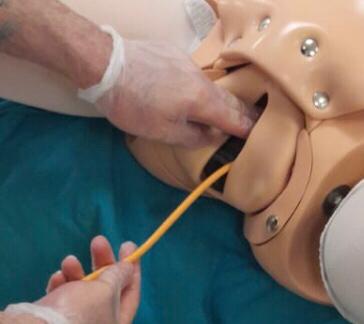


**6.** A 16 F urinary catheter was placed in the uterine cavity under the guidance of abdominal ultrasonography, and the balloon was inflated to 30 cc localized to the cesarean scar, and tamponade was applied, and this catheter was removed at the end of 24 hours.
